# Reliability and Validity of the Neuropsychiatric Inventory Questionnaire in Dyads With Dementia at Hospitalization

**DOI:** 10.1093/geroni/igab046.1450

**Published:** 2021-12-17

**Authors:** Barbara Resnick

**Affiliations:** University of Maryland School of Nursing, Baltimore, Maryland, United States

## Abstract

This study expanded on the limited psychometric testing of the Neuropsychiatric Inventory-Questionnaire (NPI-Q), and extended testing to include hospitalized persons with dementia upon admission to the hospital, with reports from family caregivers. Using data from 318 dyads in the ongoing Fam-FFC trial, a Rasch analysis was conducted. Most patients were female (62%), non-Hispanic (98%), and Black (50%) with a mean age of 81.62 (SD=8.43). There was evidence of internal consistency for all subscales (behavior, severity, caregiver distress); a DIF analysis showed invariance across race and gender. The items on the NPI-Q fit with each subscale. Hypothesis testing showed a significant association between the AD8 (F=30.04, p=.001) and MoCA (F= 5.05, p=.03) with behaviors; the AD8 (F =27.91, p=.001) and MoCA (F = 6.65, p=.01) with severity; and the AD8 (F = 29.23, p=.001) with caregiver distress. Findings provide support for the NPI-Q use in persons with dementia during acute illness.

